# Supplementary material for: Understanding the psychological experiences of loneliness in later life: qualitative protocol to inform technology development
Source: BMJ Open. 2023 Jun 19;13(6):e072420. doi: 10.1136/bmjopen-2023-072420 (PMC10314543; doi:10.1136/bmjopen-2023-072420)
Supplement: Author's manuscript [file bmjopen-2023-072420.draft_revisions.pdf]

BMJ Open

BMJ Open is committed to open peer review. As part of this commitment we make the peer review history of every article we publish publicly available.

When an article is published we post the peer reviewers' comments and the authors' responses online. We also post the versions of the paper that were used during peer review. These are the versions that the peer review comments apply to.

The versions of the paper that follow are the versions that were submitted during the peer review process. They are not the versions of record or the final published versions. They should not be cited or distributed as the published version of this manuscript.

BMJ Open is an open access journal and the full, final, typeset and author-corrected version of record of the manuscript is available on our site with no access controls, subscription charges or pay-per-view fees (http://bmjopen.bmj.com).

If you have any questions on BMJ Open's open peer review process please email info.bmjopen@bmj.com

BMJ Open

Understanding the Psychological Experiences of Loneliness in Later Life: Qualitative Protocol to Inform Technology Development

Journal:	*BMJ Open*
Manuscript ID	bmjopen-2023-072420
Article Type:	Protocol
Date Submitted by the Author:	02-Feb-2023

Complete List of Authors:	Rees, Jessica; King's College London, Institute of Gerontology<br>Tinker, Anthea; King's College London, Gerontology<br>Matcham, Faith; University of Sussex, School of Psychology<br>Probst, Freya ; King's College London, Department of Engineering<br>Antonelli, Michela ; King's College London School of Biomedical Engineering and Imaging Sciences,<br>Wang, Xi; University of Chester, Department of Physical, Mathematical and Engineering Sciences<br>Ourselin, Sebastien; King's College London, School of Biomedical Engineering & Imaging Sciences<br>Shi, Yu; University of Chester, Department of Physical, Mathematical and Engineering Sciences<br>Liu, Wei ; King's College London, Department of Engineering

Keywords:	GERIATRIC MEDICINE, Protocols & guidelines < HEALTH SERVICES ADMINISTRATION & MANAGEMENT, Public health < INFECTIOUS DISEASES, MENTAL HEALTH, QUALITATIVE RESEARCH

SCHOLARONE™
Manuscripts

Understanding the Psychological Experiences of Loneliness in Later Life: Qualitative Protocol to Inform Technology Development

Jessica Rees, PhD*[1], Anthea Tinker, PhD[1], Faith Matcham, PhD[2], Freya Probst, PhD[5], Sebastien Ourselin, PhD[3], Michela Antonelli, PhD[3], Xi Wang, PhD[4], Yu Shi, PhD[4] and Wei Liu, PhD[5].

[1] Department of Global Health and Social Medicine, King's College London, London, UK.

[2] School of Psychology, University of Sussex, Falmer, UK.

[3] School of Biomedical Engineering & Imaging Sciences, King's College London, London, UK.

[4] Department of Physical, Mathematical and Engineering Sciences, University of Chester, Chester, UK.

[5] Department of Engineering, King's College London, London, UK.

Correspondence to: Jessica Rees, Institute of Gerontology, Department of Global Health and Social Medicine, King's College London, UK. Email: Jessica.rees@kcl.ac.uk

Funding statement

This work is supported by the UK Engineering and Physical Sciences Research Council (ESPRC) and the National Institute of Health and Care Research (NIHR) (grant number: EP/W031434/1; EP/W031442/1).

Author contributions

JR led in drafting the protocol report for publication. AT and FM led the design of the methodology for data collection, with SO, YS and WL providing feedback on topic guide. WL led the overall supervision of the project. All authors have approved the final protocol for publication.

Acknowledgments

This research was reviewed by a diverse multicultural Patient and Public Involvement group who have been specially trained to advise on research proposals and documentation through the Race, Ethnicity And Diversity group (READ): a free, confidential service in England provided by the National Institute for Health Research Maudsley Biomedical Research Centre via King's College London and South London and Maudsley NHS Foundation Trust.

Competing interests: None declared.

For peer review only

Abstract

Objectives

Loneliness is a public health issue impacting the health and welling of older adults. This protocol focuses on understanding the psychological experiences of loneliness in later life to inform technology development as part of the 'Design for health ageing: a smart system to detect loneliness in older people' (DELONELINESS) study.

Methods and analysis

Data will be collected from semi-structured interviews with up to sixty people over the age of 65 on their experiences of loneliness and preferences for sensor-based technologies. The interviews will be audio-recorded, transcribed, and analysed using a thematic codebook approach on NVIVO software.

Ethics and dissemination

This study has received ethical approval by Research Ethics Committee's at King's College London (reference number: LRS/DP-21/22-33376) and the University of Sussex (reference number: ER/JH878/1). All participants will be required to provide informed consent. Results will be used to inform technology development within the DELONELINESS study and will be disseminated in peer-reviewed publications and conferences.

Strengths and limitations

- This study forms part of a wider project that will collectively develop a smart system to identify the state of loneliness in older adults.
- A strength of this study is the large sample which we will use to stratify experiences of loneliness in later life which correlate with technology preferences.
- This paper presents a project specific model of loneliness developed from the existing literature which will be iterated following data analysis.
- One limitation of this study is that conceptualisations of loneliness may exclude people from taking part in interviews.
- Findings from this research will provide greater breadth to understanding how people with varied experienced of loneliness in later life engage with technology including the identification of barriers and facilitators to technology uptake.

Keywords: Loneliness, Older Adults, Technology, Qualitative, Psychology.

Word count: 3400/4000 words

For peer review only

Introduction

Loneliness- definition, impact and prevalence

Loneliness has been defined as “an unwelcome feeling of lack or loss of companionship” (1). This is based on one theoretical framework for loneliness, where a person experiences a ‘mismatch’ (or cognitive discrepancy) between actual and desired levels of social contact or quality of relationships (2). The subjective nature of loneliness means it is experienced and felt differently by each individual. This is distinct from the concept of social isolation, which quantifies the number of connections in a person’s social network. Social isolation is most closely aligned with social loneliness, defined as an objective number of social contacts and connections (3). Emotional loneliness, which can also be relationship-specific, refers to the lack or loss of meaningful, good quality relationships (4). More recent conceptualisations have also described existential loneliness as a sense of separateness from others (5).

Loneliness is a rising public health issue (6) with detrimental impacts on an individual’s health (5). Older adults experience both physical and psychological changes as a result of loneliness (7). There is consistent evidence of the impact of loneliness on important health outcomes such incidence of cardiovascular disease (8), risk of dementia (9), and multi-morbidity (10) leading to increased mortality (11). Loneliness is an unpleasant experience (2) associated with negative emotions such as worry and sadness (5). As such, the bidirectional nature of loneliness and mental health is well known. Specifically conditions such as depression and anxiety, and symptoms such as sleep difficulties, eating or substance use disorders and suicidal ideation are more frequently reported in people experiencing loneliness (12).

Loneliness is a common experience in later life, with one in four people over the age of 65 experiencing moderate loneliness in high-income countries (13). In the UK, this translates to 1.4 million older adults experiencing loneliness often (14). Those at greater risk of feeling lonely include widowed older homeowners who live alone with a long-term health condition (15). The prevalence of loneliness in older adults reportedly increased from 26% to 32% after the first three months of the COVID-19 pandemic (16) as social distancing restrictions changed social networks and support (17).

Experiences of loneliness

Qualitative research provides useful insights into the nuanced, complex, and multi-faceted nature of loneliness. A meta-synthesis of how older adults experience and manage loneliness described how interpersonal relationships are linked with negative emotions such as helplessness, sadness, grief, disappointment (18). Loss is an important aspect of loneliness for older adults, a factor which has been outlined in the Social Relationship Expectation framework (19). Loneliness has also been described as an ‘unspoken and trivialised’ experience due to stigma which is enhanced by public discourse around ageing and health (20).

Coping strategies for loneliness employed by older adults range from prevention and action to acceptance and endurance (21). These dynamic approaches depended on whether an individual preferred coping alone or with others. Those who addressed loneliness with others were more likely to engage with services and social activities. This has implications for services to identify and provide support for individuals experiencing loneliness yet prefer to cope alone. On an individual levels, cognitive strategies (such as acceptance) can facilitate management of negative feelings associated with loneliness (22). Emotional regulation strategies for loneliness have parallels with psychological distress, although the ability to change one’s thinking about a situation (i.e. cognitive reappraisal) has been found to be low in people experiencing loneliness (23).

For peer review only - http://bmjopen.bmj.com/site/about/guidelines.xhtml

Risk factors for loneliness

There is a vast literature on the risk factors or predictors of loneliness for older adults. Categories include sociodemographic factors (e.g. age, gender, income), psychological attributes (e.g. neuroticism, self-efficacy), social resources (e.g. social contacts, marital status) and health attributes (e.g. health/functional status) (24,25). Researchers have sought to use such factors to develop profiles of individuals most at risk of loneliness (26,27). For example, older adults who are not married/widowed, those with limited social contact/support, and have poor self-report health.

Psychological factors relating to behaviours, feelings, thoughts and attitudes (28) are also relevant to the categorisation of loneliness. These include an individual's attribution style (i.e. internal or external explanations for life events), coping style (i.e. problem or emotion focused), personality characteristics (i.e. neuroticism, early life experiences) and self-esteem/efficacy (i.e. belief in own ability to succeed in social situations) (12). Lower loneliness levels have been found in people with extroversion, positive mental wellbeing, informal social contacts and where spouse is a close confidant (29). These studies are important to understand how we might measure loneliness and to increase our understanding of interacting effects (30).

Technology and loneliness

To develop effective interventions, it is important for future research to consider context-specific predictors for the dimensions of loneliness which map onto interacting explanations for behaviour (31). There is growing interest in the use of technology to detect loneliness. This is based on the principle of early intervention to prevent further physical or mental decline. Previous research has measured daily life patterns to detect loneliness using either smart-based methods (using ambient sensors to measure motion and touch) or smartphone and wearable-based sensors (32). These include mobile phone use, time spent in/out of home, sleep habits, mobility patterns, proximity sensing and conversation activity.

A recent systematic review identified seven studies which used sensor-based technologies (smart-homes) to detect or predict feelings of loneliness in older adults (33). Digital phenotypes of behaviour or daily activities can be monitored, then algorithms can be used to infer loneliness by deriving behaviour patterns from data. Such approaches have been described as "promising research path for overcoming loneliness"(33, p.10) however important ethical and privacy issues still remain to be addressed in future work.

To develop detection methods using machine learning techniques, previous research used a detailed understanding of the psycho-physiological signs and symptoms of stress (34). By further exploring the experiences of loneliness in later life, we aim to develop a context-specific understanding of the psychosocial parameters of loneliness which will inform future technology development.

This protocol

This qualitative study explores the psychological experiences of older adults and associated preferences for technology useability and engagement. Our findings will inform the development of a smart monitoring and communication system with multifunctional electronics built into textiles used as wearables and home furniture to measure loneliness levels in older people. Critical to the success of developing a novel smart monitoring system is involving end-users in the development and design of the system.

For peer review only - http://bmjopen.bmj.com/site/about/guidelines.xhtml

To gather feedback about the suitability of a smart system to detect loneliness, we aim to develop a holistic perspective of the psychological experiences of loneliness in later life, and how this relates to willingness to interact with sensor-based technologies. Our specific objectives include:

- To explore older people's experience of loneliness across a number of psychological and social parameters and behaviours.
- To describe the context and circumstance in which a smart system might be most useful and useable in an elderly population.
- To identify the most meaningful way to relaying data collected by the smart system back to individuals, their family or health and social care providers.

This study is funded by the Engineering and Physical Sciences Research Council and National Institute for Health and Care Research (grant number: EP/W031434/1;EP/W031442/1).

In Figure 1, we outline the DELONELINESS study conceptualisation of loneliness and model for the psychological aspects of loneliness in later life. We aim to analyse these factors to understand what works for who in which circumstances relating to a) The experiences of loneliness in later life and b) The preferences of older adults for sensor based technologies to detect and predict feelings of loneliness.

For peer review only

For peer review only - http://bmjopen.bmj.com/site/about/guidelines.xhtml

Figure 1: A evidence-based conceptual model of loneliness

[INSERT FIGURE 1]

Methods and analysis

Due to the subjective nature of loneliness (5), we will use a qualitative approach to explore the psychosocial parameters of loneliness which are associated with technology preferences in later life. Data will be collected using semi-structured interviews with people over the age of 65. To describe and stratify a range of experiences, we will interview up to 60 individuals with lived experience of loneliness in later life. The discussions will be semi-structured to encourage participants to explore ideas and perspectives, whilst remaining focused on the research aims. Analysis of interview data will form the basis of recommendations to develop a smart system to monitor loneliness. Data collection for this study began in October 2022 and will end in August 2023.

Recruitment

Participants will be eligible to take part in interviews if they are over the age of 65, self-identify as having experienced loneliness since reaching the age of 65, are able to give informed consent (i.e. no cognitive impairment or dementia), and speak English at a level sufficient for participation. We will purposively sample based on age, gender, accommodation type (own home, rented, sheltered accommodation) and level of digital ability. The latter will be assessed indirectly based on participants communication preferences (i.e. telephone verses email).

Study information will be sent to participants from two previous research projects who have provided consent to be contacted for future research. Firstly, participants from the Remote Assessment of Disease and Relapse (RADAR) programme which aims to examine whether wearable technology can improve symptom measurement in long-term conditions. Secondly, participants from PROTECT which is a research project investigating precursors to dementia in an ageing population. We will also share study information via national newsletters sent by email from the Housing Learning and Improvement Network, who provide specialist housing and care solutions for older adults. Finally, we will share online advertisements on research participation websites such as MQ Mental Health. For all recruitment sources, potential participants will be provided with the contact information of the research team and invited to contact us if they are interested in finding out more information. Written informed consent will be obtained from all participants prior to data collection. We will be explicit about the focus on loneliness in all recruitment material (35).

Data collection

Questionnaire data

We will ask socio-demographic information such as age, gender, marital status, ethnicity, accommodation type, and educational qualifications. Using a General Dara Protection Regulation compliant survey software system (Qualtrics), the researcher will enter verbal responses from participants. To develop a broad understanding of factors related to loneliness and technology preferences, we will record details of participants social environment (number of people seen in a typical week) and medical history (history of depression, physical activity, average sleep, details of any illnesses or disabilities).

We followed specific guidance on selecting scales to measure loneliness in later life (36). To develop our understanding on the conceptualisations of loneliness, participants will verbally provide answers

For peer review only - http://bmjopen.bmj.com/site/about/guidelines.xhtml

to two recommended loneliness measures. In addition, we will ask about mental health and service use. Details of items and scoring are provided below.

The De Jong Gierveld Loneliness (4)

Designed for use in older adult populations (3) we will use the 11-Item Scale which is a valid and reliable measure of social loneliness (5 items) and emotional loneliness (6 items). Positively worded items (All of the time, Often, Some of the time) are counted for emotional loneliness statements, while negatively worded items (None of the time, Rarely, Some of the time) are counted for social loneliness statements. The total loneliness score is calculated by taking the sum of the emotional loneliness score and the social loneliness score, which can be categorised into four levels: not lonely (score 0, 1 or 2), moderate lonely (score 3 through 8), severe lonely (score 9 or 10), and very severe lonely (score 11).

The Office for National Statistics recommended measures(37)

Following recommendations of the Office for National Statistics, we will use three questions from the University of California, Los Angeles (UCLA) three-item loneliness scale (38) relating to companionship, feeling left out and feeling isolated from others. Response options include "hardly ever or ever", "some of the time" and "often" scored as 1, 2, and 3, respectively. Scores can be added together and grouped into two categories (39): lonely (scores 6 through 9) and not lonely (scores 3 through 5). We then ask a direct measure of loneliness with response options of "often/always", "some of the time", "occasionally", "never".

The Patient Health Questionnaire Anxiety and Depression (40)

We will use the 4-item scale (41) of this reliable and valid consisting of two core depression items and two core anxiety items. Participant report how symptoms have affected them over the past two weeks with response options of "not at all", "several days", "more than half the days", and "nearly every day", scored as 0, 1, 2, and 3, respectively. Total score is determined by adding together the scores of each of the 4 items. Scores are rated as normal (0-2), mild (3-5), moderate (6-8), and severe (9-12). Total score ≥3 for first 2 questions suggests anxiety. Total score ≥3 for last 2 questions suggests depression.

The Modified Client Services Receipt Inventory for healthcare service use (42)

The Client Service Receipt inventory is a widely used health resource measurement tool (43). We will use the modified version to record consultations with healthcare practitioners and hospital admissions, and medication taken (within three months). We will also record help received as consequence of health problems (childcare, personal care, help in/outside home).

Qualitative interview

To increase accessibility for those who cannot travel or do not have access to the internet, interviews will be held either in-person (at participants homes/in university offices) or remotely (via Microsoft Teams video call or telephone call). The format of interviews will depend on participant need and preference. Interviews will last no longer than 120 minutes, with opportunities for comfort breaks provided. Participants will receive a £30 voucher for taking part in the interviews, to represent the time dedicated to providing data.

The interview guide will include specific questions on participants personal experience of loneliness including definition, correlates, precursors and support received. We developed the topic guide with a multi-disciplinary team with expertise in psychology, gerontology, product design, smart

For peer review only - http://bmjopen.bmj.com/site/about/guidelines.xhtml

For peer review only

composite materials and artificial intelligence. In line with recent guidelines on interviewing older adults about loneliness (35) we will begin the interview using a third-person approach by asking participants how they define the term 'loneliness'. We will then move onto exploring participants own personal experiences. To co-construct narratives of loneliness we will prompt participants about their responses to the loneliness items and asked to reflect and expand on their experiences.

The second part of the interview will explore the role of technology in measuring loneliness. To inform product design, we will ask for descriptions of participants living environment, daily routine, existing use of technologies for health and preferences for wearables or sensors in furniture or clothing. The final section will focus on the use of data, where we ask participants for their preferences of how data will be feedback to older adults, circumstances where data is shared with family members and/or health and social care professionals, and thoughts/feelings about being alerted to risk of becoming loneliness.

The interviewer will always end the interview of a positive note (35). If a sensitive topic is disclosed shortly before the end of the interview, the researcher will check in with the participant and go back to a positive topic previously discussed. If the researcher has safety concerns, they will make it clear whether the participant should expect a follow-up email or telephone call. Interviews will be audio-recorded and transcribed verbatim by an external company.

Data analysis

Analysis will be informed by the theory gleaning stage of realist methodology which aims to understand 'what works for whom in which contexts' (44). Explanatory IF-THEN statements will be developed and tested in an iterative process. For example, IF participants experienced severe and persistent loneliness THEN usefulness of sensor was low due to perceived inability to change personal circumstances. Previous research has used realist evaluation to develop a personalised approach to intervention development in older adults due to the variety in cause and consequence of loneliness (45). Realist configurations (Context-outcome-mechanisms statements) aim to develop key clusters of properties or attributes which underpin cases (46). We will use this to develop a context-specific understanding of the psychosocial parameters of loneliness which relate to the barriers and facilitators for sensor-based technology uptake.

We will use a codebook approach to qualitative analysis which combines reflexive thematic analysis with structured early theme development (47). We will use our model of loneliness to generate initial codes based on typologies of loneliness. We will then analyse interview data coding relevant data into these topic summaries. Throughout we will use inductive approaches to review themes and define and name themes which were not present in the codebook. The process of analysis will be iterative by comparing knowledge from the literature with interviews. We will use NVIVO software to facilitate the organisation of codes during analysis.

Analysis will be undertaken by the lead author (JR). The process of familiarisation will begin at begin at the point of interviews with the checking of transcripts. Members of the research team will be consulted in regular biweekly meetings throughout data collection and analysis to discuss developing findings. The use of memo notes will be implemented throughout to record decisions on theme development to increase transparency and share findings with wider team.

Public and patient involvement

The DELONELINESS project seeks consultation from a public and patient involvement (PPI) group which includes over 70 adults with an interest in mental health research and commitment to

For peer review only - http://bmjopen.bmj.com/site/about/guidelines.xhtml

improve inclusivity by advising on race and ethnicity. In October 2022, four members of the group provided feedback on the recruitment methods outlined in this protocol. The group will be consulted throughout the project, specifically to provide feedback on preliminary findings in July 2023 and strategies for dissemination in October 2023.

Ethics and dissemination

Ethical considerations

This study has been approved by Research Ethics Committees at King's College London (reference number: LRS/DP-21/22-33376) and the University of Sussex (reference number: ER/JH878/1). The researcher conducting interviews is skilled in qualitative methods relating to sensitive topics in health and ageing and will adopt an ethical positioning of engagement and mutual respect (35). Regular clinical supervision with a qualified health psychologist will take place to support the interviewer with emotional aspects of conversations.

Time will be taken at the beginning of each interview to establish a rapport with participants and develop an understand of their own personal definition of loneliness. A risk-protocol is in place for if participants were to elicit an emotional response during interviews. With appropriate consent, the risk protocol includes recording participants GP details. In line with GDPR and data protection requirements the study has data handling and de-identification processes in place to maintain the confidentiality of data will be explained to participants involved in the interviews.

Dissemination

The findings will initially be disseminated within the study team via internal reports and meetings attending by all co-authors. The aim will be to share preliminary findings of understandings of how people with varied experienced of loneliness in later life engage with technology including the identification of barriers and facilitators to technology uptake. For academic dissemination, findings will also be published in peer-reviewed journals and shared at conferences. For public dissemination, we will share findings at meetings and events including webinars run by project partners Housing Learning Improvement Network, and through blogs published via the Campaign to End Loneliness.

References

1. Department for Digital Culture Media and Sport. A connected society: a strategy for tackling loneliness [Internet]. 2018 [cited 2022 Dec 16]. Available from: https://www.gov.uk/government/publications/a-connected-society-a-strategy-for-tackling-loneliness

2. Perlman D, Peplau LA. Toward a Social Psychology of Loneliness. 1981;

3. Maes M, Qualter P, Lodder GMA, Mund M. How (Not) to Measure Loneliness: A Review of the Eight Most Commonly Used Scales. International Journal of Environmental Research and Public Health 2022, Vol 19, Page 10816 [Internet]. 2022 Aug 30 [cited 2022 Sep 13];19(17):10816. Available from: https://www.mdpi.com/1660-4601/19/17/10816/htm

4. de Jong Gierveld J, Tilburg TV. The De Jong Gierveld short scales for emotional and social loneliness: tested on data from 7 countries in the UN generations and gender surveys. Eur J Ageing. 2010;121–30.

For peer review only - http://bmjopen.bmj.com/site/about/guidelines.xhtml

For peer review only

5. Mansfield L, Victor C, Meads C, Daykin N, Tomlinson A, Lane J, et al. A conceptual review of loneliness in adults: Qualitative evidence synthesis. Vol. 18, International Journal of Environmental Research and Public Health. MDPI; 2021.

6. Cacioppo JT, Cacioppo S. The growing problem of loneliness. The Lancet [Internet]. 2018 Feb 3 [cited 2022 Oct 20];391(10119):426. Available from: http://www.thelancet.com/article/S0140673618301429/fulltext

7. Blakely KK, Baker NR. Loneliness in Community-Dwelling, Older Adults: An Integrative Review. The Journal for Nurse Practitioners. 2022 Aug 4;

8. Courtin E, Knapp M. Social isolation, loneliness and health in old age: a scoping review. Health Soc Care Community [Internet]. 2017 May 1 [cited 2022 Sep 28];25(3):799–812. Available from: https://onlinelibrary.wiley.com/doi/full/10.1111/hsc.12311

9. Donovan NJ, Okereke OI, Vannini P, Amariglio RE, Rentz DM, Marshall GA, et al. Association of Higher Cortical Amyloid Burden With Loneliness in Cognitively Normal Older Adults. JAMA Psychiatry [Internet]. 2016 Dec 1 [cited 2022 Dec 20];73(12):1230–7. Available from: https://jamanetwork.com/journals/jamapsychiatry/fullarticle/2575729

10. Cacioppo S, Capitanio JP, Cacioppo JT. Toward a neurology of loneliness. Vol. 140, Psychological bulletin. 2014. p. 1464–504.

11. Holt-Lunstad J, Smith TB, Baker M, Harris T, Stephenson D. Loneliness and Social Isolation as Risk Factors for Mortality: A Meta-Analytic Review. Perspectives on Psychological Science. 2015 Mar 16;10(2):227–37.

12. Campaign to end loneliness. The Psychology of Loneliness: Why it matters and what we can do [Internet]. 2020 [cited 2023 Jan 19]. Available from: https://www.campaigntoendloneliness.org/wp-content/uploads/Psychology_of_Loneliness_FINAL_REPORT.pdf

13. Chawla K, Kunonga TP, Stow D, Barker R, Craig D, Hanratty B. Prevalence of loneliness amongst older people in high-income countries: A systematic review and meta-analysis. PLoS One [Internet]. 2021 Jul 1 [cited 2022 Dec 16];16(7):e0255088. Available from: https://journals.plos.org/plosone/article?id=10.1371/journal.pone.0255088

14. Age UK. All the Lonely People: Loneliness in Later Life. 2018.

15. Office for national statistics. Loneliness: What characteristics and circumstances are associated with feeling lonely? 2018;

16. Su Y, Rao W, Li M, Caron G, Arcy CD', Meng X. Prevalence of loneliness and social isolation among older adults during the COVID-19 pandemic: A systematic review and meta-analysis. Int Psychogeriatr [Internet]. 2022 [cited 2022 Dec 13];1–13. Available from: https://doi.org/10.1017/S1041610222000199

17. Vlachantoni A, Evandrou M, Falkingham J, Qin M. The Impact of Changing Social Support on Older Persons' Onset of Loneliness During the COVID-19 Pandemic in the United Kingdom. European Journal of Ageing . 2010 Sep 7;62(8):1147–59.

18. Kitzmüller G, Clancy A, Vaismoradi M, Wegener C, Bondas T. "Trapped in an Empty Waiting Room"—The Existential Human Core of Loneliness in Old Age: A Meta-Synthesis. Qual Health Res. 2018 Jan 1;28(2):213–30.

For peer review only - http://bmjopen.bmj.com/site/about/guidelines.xhtml

19. Akhter-Khan S, Prina M, Wong G, Mayston R, Li L. Understanding and Addressing Older Adults' Loneliness: The Social Relationship Expectations Framework. Perspectives on Psychological Science. 2022;

20. Malli MA, Ryan S, Maddison J, Kharicha K. Experiences and meaning of loneliness beyond age and group identity. Sociol Health Illn [Internet]. 2022 [cited 2022 Dec 13]; Available from: https://onlinelibrary.wiley.com/doi/full/10.1111/1467-9566.13539

21. Kharicha K, Manthorpe J, Iliffe S, Davies N, Walters K. Strategies employed by older people to manage loneliness: Systematic review of qualitative studies and model development. Vol. 30, International Psychogeriatrics. Cambridge University Press; 2018. p. 1767–81.

22. Kharicha K, Manthorpe J, Iliffe S, Chew-Graham CA, Cattan M, Goodman C, et al. Managing loneliness: a qualitative study of older people's views. Aging Ment Health. 2021;25(7):1206–13.

23. Tan AJ, Mancini V, Gross JJ, Goldenberg A, Badcock JC, Lim MH, et al. Loneliness Versus Distress: A Comparison of Emotion Regulation Profiles. 2022 [cited 2022 Dec 13]; Available from: https://doi.org/10.1017/bec.2022.18

24. Barjaková M, Garnero A. Risk factors for loneliness A literature review [Internet]. 2022. Available from: https://ec.europa.eu/jrc

25. Cohen-Mansfield J, Hazan H, Lerman Y, Shalom V. Correlates and predictors of loneliness in older-adults: a review of quantitative results informed by qualitative insights. Int Psychogeriatr [Internet]. 2016 Apr 1 [cited 2022 Sep 7];28(4):557–76. Available from: https://www.cambridge.org/core/journals/international-psychogeriatrics/article/abs/correlates-and-predictors-of-loneliness-in-olderadults-a-review-of-quantitative-results-informed-by-qualitative-insights/EF57448627F1EF5CFD89F83C3F188175

26. Dahlberg L, McKee KJ, Frank A, Naseer M. A systematic review of longitudinal risk factors for loneliness in older adults. Aging Ment Health [Internet]. 2022 [cited 2022 Oct 13];26(2):225–49. Available from: https://www.tandfonline.com/doi/abs/10.1080/13607863.2021.1876638

27. Hutten E, Jongen EMM, Hajema KJ, Ruiter RAC, Hamers F, Bos AER. Risk factors of loneliness across the life span. J Soc Pers Relat. 2022 May 1;39(5):1482–507.

28. Matcham F, Ali S, Hotopf M, Chalder T. Psychological correlates of fatigue in rheumatoid arthritis: A systematic review. Vol. 39, Clinical Psychology Review. Elsevier Inc.; 2015. p. 16–29.

29. Ejlskov L, Wulff J, Bøggild H, Kuh D, Stafford M. Assessing the relative importance of correlates of loneliness in later life. Gaining insight using recursive partitioning. Aging Ment Health [Internet]. 2018 Nov 2 [cited 2022 Sep 28];22(11):1486–93. Available from: https://www.tandfonline.com/doi/abs/10.1080/13607863.2017.1370690

30. Teater B, Chonody JM, Davis N. Risk and Protective Factors of Loneliness among Older Adults: The Significance of Social Isolation and Quality and Type of Contact. Soc Work Public Health. 2021;36(2):128–41.

31. Akhter-Khan S, Au R. Why Loneliness Interventions Are Unsuccessful: A Call for Precision Health. Adv Geriatr Med Res. 2020;

For peer review only

32. Qirtas MM, Zafeiridi E, Pesch D, White EB. Loneliness and Social Isolation Detection Using Passive Sensing Techniques: Scoping Review. JMIR Mhealth Uhealth [Internet]. 2022 Apr 1 [cited 2022 Sep 29];10(4). Available from: /pmc/articles/PMC9044142/

33. Latikka R, Rubio-Hernández R, Lohan ES, Rantala J, Nieto Fernández F, Laitinen A, et al. Older Adults' Loneliness, Social Isolation, and Physical Information and Communication Technology in the Era of Ambient Assisted Living: A Systematic Literature Review. J Med Internet Res 2021;23(12):e28022 https://www.jmir.org/2021/12/e28022 [Internet]. 2021 Dec 30 [cited 2022 Sep 29];23(12):e28022. Available from: https://www.jmir.org/2021/12/e28022

34. Gedam S, Paul S. A Review on Mental Stress Detection Using Wearable Sensors and Machine Learning Techniques. IEEE Access. 2021;9:84045–66.

35. Naughton-Doe R, Barke J, Manchester H, Willis P, Wigfield A. Ethical issues when interviewing older people about loneliness: reflections and recommendations for an effective methodological approach. Ageing Soc [Internet]. 2022 Sep 1 [cited 2022 Sep 7];1–19. Available from: https://www.cambridge.org/core/journals/ageing-and-society/article/ethical-issues-when-interviewing-older-people-about-loneliness-reflections-and-recommendations-for-an-effective-methodological-approach/A98A61944D6152B43DC68FAA444B3363

36. Campaign to end loneliness. Measuring your impact on loneliness in later life [Internet]. 2015 [cited 2022 Dec 16]. Available from: https://www.campaigntoendloneliness.org/wp-content/uploads/Loneliness-Measurement-Guidance1.pdf

37. Office for National Statistics. Measuring loneliness: guidance for use of the national indicators on surveys [Internet]. 2018 [cited 2022 Dec 16]. Available from: https://www.ons.gov.uk/peoplepopulationandcommunity/wellbeing/methodologies/measuringlonelinessguidanceforuseofthenationalindicatorsonsurveys

38. Hughes ME, Waite LJ, Hawkley LC, Cacioppo JT. A Short Scale for Measuring Loneliness in Large Surveys: Results From Two Population-Based Studies NIH Public Access. Finally, shifting Res Aging. 1999;26(6):655–72.

39. Steptoe A, Shankar A, Demakakos P, Wardle J. Social isolation, loneliness, and all-cause mortality in older men and women. Proc Natl Acad Sci U S A [Internet]. 2013 Apr 9 [cited 2022 Dec 16];110(15):5797–801. Available from: https://www.pnas.org/doi/abs/10.1073/pnas.1219686110

40. Kroenke K, Wu J, Yu Z, Bair MJ, Kean J, Stump T, et al. Patient health questionnaire anxiety and depression scale: Initial validation in three clinical trials. Psychosom Med [Internet]. 2016 Jul 1 [cited 2022 Dec 16];78(6):716–27. Available from: https://journals.lww.com/psychosomaticmedicine/Fulltext/2016/07000/Patient_Health_Questionnaire_Anxiety_and.10.aspx

41. Kroenke K, Spitzer RL, Williams JBW, Löwe B. An Ultra-Brief Screening Scale for Anxiety and Depression: The PHQ–4. Psychosomatics. 2009 Nov 1;50(6):613–21.

42. Beecham J, Knapp M. Costing psychiatric interventions. In: Measuring Mental Health Needs. 2nd ed. Gaskell; 2001. p. 200–24.

43. Chung CCY, Fung JLF, Lui ACY, Chan MCY, Ng YNC, Wong WHS, et al. Client Service Receipt Inventory as a standardised tool for measurement of socio-economic costs in the rare genetic

For peer review only - http://bmjopen.bmj.com/site/about/guidelines.xhtml

disease population (CSRI-Ra). Nature Scientific Reports [Internet]. 2021 [cited 2022 Dec 16];11(23837). Available from: www.nature.com/scientificreports

44. Pawson R, Tilley N. Realist evaluation . Sage, editor. 1997.

45. Day A, Phelps K, Maltby J, Palmer E, Snell K, Raine D, et al. A realist evaluation of loneliness interventions for older people. Age Ageing [Internet]. 2021 Nov 10 [cited 2022 Dec 21];50(6):2246–53. Available from: https://academic.oup.com/ageing/article/50/6/2246/6376583

46. Pawson R. Causality for beginngers [Internet]. 2008 [cited 2022 Dec 21]. Available from: http://eprints.ncrm.ac.uk/ 245/

47. Braun V, Clarke V. Reflecting on reflexive thematic analysis. Qual Res Sport Exerc Health [Internet]. 2019 Aug 8 [cited 2022 Dec 21];11(4):589–97. Available from: https://www.tandfonline.com/doi/abs/10.1080/2159676X.2019.1628806

For peer review only

For peer review only - http://bmjopen.bmj.com/site/about/guidelines.xhtml

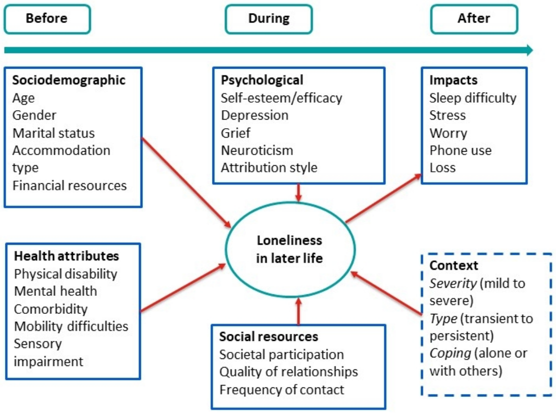

Figure 1

338x190mm (96 x 96 DPI)

BMJ Open

Understanding the Psychological Experiences of Loneliness in Later Life: Qualitative Protocol to Inform Technology Development

Journal:	*BMJ Open*
Manuscript ID	bmjopen-2023-072420.R1
Article Type:	Protocol
Date Submitted by the Author:	25-Apr-2023

Complete List of Authors:	Rees, Jessica; King's College London, Institute of Gerontology<br>Liu, Wei ; King's College London, Department of Engineering<br>Ourselin, Sebastien; King's College London, School of Biomedical Engineering & Imaging Sciences<br>Shi, Yu; University of Chester, Department of Physical, Mathematical and Engineering Sciences<br>Probst, Freya ; King's College London, Department of Engineering<br>Antonelli, Michela ; King's College London School of Biomedical Engineering and Imaging Sciences,<br>Tinker, Anthea; King's College London, Gerontology<br>Matcham, Faith; University of Sussex, School of Psychology

**Primary Subject Heading**:	Geriatric medicine
Secondary Subject Heading:	Research methods
Keywords:	GERIATRIC MEDICINE, Protocols & guidelines < HEALTH SERVICES ADMINISTRATION & MANAGEMENT, Public health < INFECTIOUS DISEASES, MENTAL HEALTH, QUALITATIVE RESEARCH

SCHOLARONE™
Manuscripts

Understanding the Psychological Experiences of Loneliness in Later Life: Qualitative Protocol to Inform Technology Development

Jessica Rees, PhD*[1], Wei Liu, PhD*[2], Sebastien Ourselin, PhD[3], Yu Shi, PhD[4], Freya Probst, PhD[2], Michela Antonelli, PhD[3], Anthea Tinker, PhD[1], and Faith Matcham, PhD[5].

[1] Department of Global Health and Social Medicine, King's College London, London, UK.

[2] Department of Engineering, King's College London, London, UK.

[3] School of Biomedical Engineering & Imaging Sciences, King's College London, London, UK.

[4] Department of Physical, Mathematical and Engineering Sciences, University of Chester, Chester, UK.

[5] School of Psychology, University of Sussex, Falmer, UK.

Correspondence to: Jessica Rees, Institute of Gerontology, Department of Global Health and Social Medicine, King's College London, UK. Email: Jessica.rees@kcl.ac.uk and Wei Liu, Department of Engineering, King's College London, UK. Email: wei.liu@kcl.ac.uk

Abstract

Objectives

Loneliness is a public health issue impacting the health and wellbeing of older adults. This protocol focuses on understanding the psychological experiences of loneliness in later life to inform technology development as part of the 'Design for health ageing: a smart system to detect loneliness in older people' (DELONELINESS) study.

Methods and analysis

Data will be collected from semi-structured interviews with up to sixty people over the age of 65 on their experiences of loneliness and preferences for sensor-based technologies. The interviews will be audio-recorded, transcribed, and analysed using a thematic codebook approach on NVIVO software.

Ethics and dissemination

This study has received ethical approval by Research Ethics Committee's at King's College London (reference number: LRS/DP-21/22-33376) and the University of Sussex (reference number: ER/JH878/1). All participants will be required to provide informed consent. Results will be used to inform technology development within the DELONELINESS study and will be disseminated in peer-reviewed publications and conferences.

Strengths and limitations

- A strength of this study is the large sample (n=60) which we will use to stratify experiences of loneliness in later life which correlate with technology preferences.
- We have integrated patient and public involvement into the design and conduct of our study.
- The data collection methods presented in this protocol are inclusive to facilitate the participation of older adults based on technology preferences.
- One limitation of this study is that conceptualisations of loneliness may exclude people from taking part in interviews.
-

For peer review only - http://bmjopen.bmj.com/site/about/guidelines.xhtml

Keywords: Loneliness, Older Adults, Technology, Qualitative, Psychology.

Word count: 3500/4000 words

For peer review only

For peer review only - http://bmjopen.bmj.com/site/about/guidelines.xhtml

Introduction

Loneliness- definition, impact and prevalence

Loneliness has been defined as "an unwelcome feeling of lack or loss of companionship" (1). This is based on one theoretical framework for loneliness, where a person experiences a 'mismatch' (or cognitive discrepancy) between actual and desired levels of social contact or quality of relationships (2). The subjective nature of loneliness means it is experienced and felt differently by each individual. This is distinct from the concept of social isolation, which quantifies the number of connections in a person's social network. Social isolation is most closely aligned with social loneliness, defined as an objective number of social contacts and connections (3). Emotional loneliness, which can also be relationship-specific, refers to the lack or loss of meaningful, good quality relationships (4). More recent conceptualisations have also described existential loneliness as a sense of separateness from others (5).

Loneliness is a rising public health issue (6) with detrimental impacts on an individual's health (5). Older adults experience both physical and psychological changes as a result of loneliness (7). There is consistent evidence of the impact of loneliness on important health outcomes such incidence of cardiovascular disease (8), risk of dementia (9), and multi-morbidity (10) leading to increased mortality (11). Loneliness is an unpleasant experience (2) associated with negative emotions such as worry and sadness (5). As such, the bidirectional nature of loneliness and mental health is well known. Specifically conditions such as depression and anxiety, and symptoms such as sleep difficulties, eating or substance use disorders and suicidal ideation are more frequently reported in people experiencing loneliness (12).

Loneliness is a common experience in later life, with one in four people over the age of 65 experiencing moderate loneliness in high-income countries (13). In the UK, this translates to 1.4 million older adults experiencing loneliness often (14). Those at greater risk of feeling lonely include widowed older homeowners who live alone with a long-term health condition (15). The prevalence of loneliness in older adults reportedly increased from 26% to 32% after the first three months of the COVID-19 pandemic (16) as social distancing restrictions changed social networks and support (17).

Experiences of loneliness

Qualitative research provides useful insights into the nuanced, complex, and multi-faceted nature of loneliness. A meta-synthesis of how older adults experience and manage loneliness described how interpersonal relationships are linked with negative emotions such as helplessness, sadness, grief, disappointment (18). Loss is an important aspect of loneliness for older adults, a factor which has been outlined in the Social Relationship Expectation framework (19). Loneliness has also been described as an 'unspoken and trivialised' experience due to stigma which is enhanced by public discourse around ageing and health (20).

Coping strategies for loneliness employed by older adults range from prevention and action to acceptance and endurance (21). These dynamic approaches depended on whether an individual preferred coping alone or with others. Those who addressed loneliness with others were more likely to engage with services and social activities. This has implications for services to identify and provide support for individuals experiencing loneliness yet prefer to cope alone. On an individual levels, cognitive strategies (such as acceptance) can facilitate management of negative feelings associated with loneliness (22). Emotional regulation strategies for loneliness have parallels with psychological distress, although the ability to change one's thinking about a situation (i.e. cognitive reappraisal) has been found to be low in people experiencing loneliness (23).

For peer review only - http://bmjopen.bmj.com/site/about/guidelines.xhtml

For peer review only

Risk factors for loneliness

There is a vast literature on the risk factors or predictors of loneliness for older adults. Categories include sociodemographic factors (e.g. age, gender, income), psychological attributes (e.g. neuroticism, self-efficacy), social resources (e.g. social contacts, marital status) and health attributes (e.g. health/functional status) (24,25). Researchers have sought to use such factors to develop profiles of individuals most at risk of loneliness (26,27). For example, older adults who are not married/widowed, those with limited social contact/support, and have poor self-report health.

Psychological factors relating to behaviours, feelings, thoughts and attitudes (28) are also relevant to the categorisation of loneliness. These include an individual's attribution style (i.e. internal or external explanations for life events), coping style (i.e. problem or emotion focused), personality characteristics (i.e. neuroticism, early life experiences) and self-esteem/efficacy (i.e. belief in own ability to succeed in social situations) (12). Lower loneliness levels have been found in people with extroversion, positive mental wellbeing, informal social contacts and where spouse is a close confidant (29). These studies are important to understand how we might measure loneliness and to increase our understanding of interacting effects (30).

Technology and loneliness

To develop effective interventions, it is important for future research to consider context-specific predictors for the dimensions of loneliness which map onto interacting explanations for behaviour (31). There is growing interest in the use of technology to detect loneliness. This is based on the principle of early intervention to prevent further physical or mental decline. Previous research has measured daily life patterns to detect loneliness using either smart-based methods (using ambient sensors to measure motion and touch) or smartphone and wearable-based sensors (32). These include mobile phone use, time spent in/out of home, sleep habits, mobility patterns, proximity sensing and conversation activity.

A recent systematic review identified seven studies which used sensor-based technologies (smart-homes) to detect or predict feelings of loneliness in older adults (33). Digital phenotypes of behaviour or daily activities can be monitored, then algorithms can be used to infer loneliness by deriving behaviour patterns from data. Such approaches have been described as "promising research path for overcoming loneliness"(33, p.10) however important ethical and privacy issues still remain to be addressed in future work.

To develop detection methods using machine learning techniques, previous research used a detailed understanding of the psycho-physiological signs and symptoms of stress (34). By further exploring the experiences of loneliness in later life, we aim to develop a context-specific understanding of the psychosocial parameters of loneliness which will inform future technology development.

This protocol

This qualitative study explores the psychological experiences of loneliness in older adults and associated preferences for technology useability and engagement. Our findings will inform the development of a smart monitoring and communication system with multifunctional electronics built into textiles used as wearables and home furniture to measure loneliness levels in older people. Critical to the success of developing a novel smart monitoring system is involving end-users in the development and design of the system.

For peer review only - http://bmjopen.bmj.com/site/about/guidelines.xhtml

To gather feedback about the suitability of a smart system to detect loneliness, we aim to develop a holistic perspective of the psychological experiences of loneliness in later life, and how this relates to willingness to interact with sensor-based technologies. Our specific objectives include:

- To explore older people's experience of loneliness across a number of psychological and social parameters and behaviours.
- To describe the context and circumstance in which a smart system might be most useful and useable in an elderly population.
- To identify the most meaningful way to relay data collected by the smart system back to individuals, their family or health and social care providers.

This study is funded by the Engineering and Physical Sciences Research Council and National Institute for Health and Care Research (grant number: EP/W031434/1;EP/W031442/1).

In Figure 1, we outline the DELONELINESS study conceptualisation of loneliness and model for the psychological aspects of loneliness in later life. We aim to analyse these factors to understand what works for who in which circumstances relating to a) The experiences of loneliness in later life and b) The preferences of older adults for sensor based technologies to detect and predict feelings of loneliness.

For peer review only

For peer review only - http://bmjopen.bmj.com/site/about/guidelines.xhtml

Figure 1: A evidence-based conceptual model of loneliness

[INSERT FIGURE 1]

Methods and analysis

Due to the subjective nature of loneliness (5), we will use a qualitative approach to explore the psychosocial parameters of loneliness which are associated with technology preferences in later life. Data will be collected using semi-structured interviews with people over the age of 65. To describe and stratify a range of experiences, we will interview up to 60 individuals with lived experience of loneliness in later life. The discussions will be semi-structured to encourage participants to explore ideas and perspectives, whilst remaining focused on the research aims. Analysis of interview data will form the basis of recommendations to develop a smart system to monitor loneliness. Data collection for this study began in October 2022 and will end in August 2023.

Recruitment

Participants will be eligible to take part in interviews if they are over the age of 65, self-identify as having experienced loneliness since reaching the age of 65, are able to give informed consent (i.e. no cognitive impairment or dementia), and speak English at a level sufficient for participation. We will purposively sample based on age, gender, accommodation type (own home, rented, sheltered accommodation) and level of digital ability. The latter will be assessed indirectly based on participants communication preferences (i.e. telephone verses email).

Study information will be sent to participants from two previous research projects who have provided consent to be contacted for future research. Firstly, participants from the Remote Assessment of Disease and Relapse – Major Depressive Disorder (RADAR-MDD) programme which aims to examine whether wearable technology can improve symptom measurement in long-term conditions (35). Secondly, participants from PROTECT which is a research project investigating precursors to dementia in an ageing population. We will also share study information via national newsletters sent by email from the Housing Learning and Improvement Network, who provide specialist housing and care solutions for older adults. Finally, we will share online advertisements on research participation websites such as MQ Mental Health. For all recruitment sources, potential participants will be provided with the contact information of the research team and invited to contact us if they are interested in finding out more information. Written informed consent will be obtained from all participants prior to data collection. We will be explicit about the focus on loneliness in all recruitment material (36).

Data collection

Questionnaire data

We will ask socio-demographic information such as age, gender, marital status, ethnicity, accommodation type, and educational qualifications. Using a General Dara Protection Regulation compliant survey software system (Qualtrics), the researcher will enter verbal responses from participants. To develop a broad understanding of factors related to loneliness and technology preferences, we will record details of participants social environment (number of people seen in a typical week) and medical history (history of depression, physical activity, average sleep, details of any illnesses or disabilities).

We followed specific guidance on selecting scales to measure loneliness in later life (37). To develop our understanding on the conceptualisations of loneliness, participants will verbally provide answers

For peer review only - http://bmjopen.bmj.com/site/about/guidelines.xhtml

to two recommended loneliness measures. Both measures will be used to validate participants self-identification of loneliness experiences and indicate level of loneliness severity at the time of interview. In addition, we will ask about mental health and service use. Details of items and scoring are provided below.

The De Jong Gierveld Loneliness (4)

Designed for use in older adult populations (3) we will use the 11-Item Scale which is a valid and reliable measure of social loneliness (5 items) and emotional loneliness (6 items). Positively worded items (All of the time, Often, Some of the time) are counted for emotional loneliness statements, while negatively worded items (None of the time, Rarely, Some of the time) are counted for social loneliness statements. The total loneliness score is calculated by taking the sum of the emotional loneliness score and the social loneliness score, which can be categorised into four levels: not lonely (score 0, 1 or 2), moderate lonely (score 3 through 8), severe lonely (score 9 or 10), and very severe lonely (score 11).

The Office for National Statistics recommended measures(38)

Following recommendations of the Office for National Statistics, we will use three questions from the University of California, Los Angeles (UCLA) three-item loneliness scale (39) relating to companionship, feeling left out and feeling isolated from others. Response options include "hardly ever or ever", "some of the time" and "often" scored as 1, 2, and 3, respectively. Scores can be added together and grouped into two categories (40): lonely (scores 6 through 9) and not lonely (scores 3 through 5). We then ask a direct measure of loneliness with response options of "often/always", "some of the time", "occasionally", "never".

The Patient Health Questionnaire Anxiety and Depression (41)

We will use the 4-item scale (42) of this reliable and valid consisting of two core depression items and two core anxiety items. Participant report how symptoms have affected them over the past two weeks with response options of "not at all", "several days", "more than half the days", and "nearly every day", scored as 0, 1, 2, and 3, respectively. Total score is determined by adding together the scores of each of the 4 items. Scores are rated as normal (0-2), mild (3-5), moderate (6-8), and severe (9-12). Total score ≥3 for first 2 questions suggests anxiety. Total score ≥3 for last 2 questions suggests depression.

The Modified Client Services Receipt Inventory for healthcare service use (43)

The Client Service Receipt inventory is a widely used health resource measurement tool (44). We will use the modified version to record consultations with healthcare practitioners and hospital admissions, and medication taken (within three months). We will also record help received as consequence of health problems (childcare, personal care, help in/outside home).

Qualitative interview

To increase accessibility for those who cannot travel or do not have access to the internet, interviews will be held either in-person (at participants homes/in university offices) or remotely (via Microsoft Teams video call or telephone call). The format of interviews will depend on participant need and preference. Interviews will last no longer than 120 minutes, with opportunities for comfort breaks provided. Participants will receive a £30 voucher for taking part in the interviews, to represent the time dedicated to providing data.

For peer review only - http://bmjopen.bmj.com/site/about/guidelines.xhtml

The interview guide (see supplementary material) will include specific questions on participants personal experience of loneliness including definition, correlates, precursors and support received. We developed the topic guide with a multi-disciplinary team with expertise in psychology, gerontology, product design, smart composite materials and artificial intelligence. In line with recent guidelines on interviewing older adults about loneliness (36) we will begin the interview using a third-person approach by asking participants how they define the term 'loneliness'. We will then move onto exploring participants own personal experiences. To co-construct narratives of loneliness we will prompt participants about their responses to the loneliness items and asked to reflect and expand on their experiences.

The second part of the interview will explore the role of technology in measuring loneliness. To inform product design, we will ask for descriptions of participants living environment, daily routine, existing use of technologies for health and preferences for wearables or sensors in furniture or clothing. The final section will focus on the use of data, where we ask participants for their preferences of how data will be feedback to older adults, circumstances where data is shared with family members and/or health and social care professionals, and thoughts/feelings about being alerted to risk of becoming loneliness.

The interviewer will always end the interview of a positive note (36). If a sensitive topic is disclosed shortly before the end of the interview, the researcher will check in with the participant and go back to a positive topic previously discussed. If the researcher has safety concerns, they will make it clear whether the participant should expect a follow-up email or telephone call. Interviews will be audio-recorded and transcribed verbatim by an external company.

Data analysis

Analysis will be informed by the theory gleaning stage of realist methodology which aims to understand 'what works for whom in which contexts' (45). Explanatory IF-THEN statements will be developed and tested in an iterative process. For example, IF participants experienced severe and persistent loneliness THEN usefulness of sensor was low due to perceived inability to change personal circumstances. Previous research has used realist evaluation to develop a personalised approach to intervention development in older adults due to the variety in cause and consequence of loneliness (46). Realist configurations (Context-outcome-mechanisms statements) aim to develop key clusters of properties or attributes which underpin cases (47). We will use this to develop a context-specific understanding of the psychosocial parameters of loneliness which relate to the barriers and facilitators for sensor-based technology uptake.

We will use a codebook approach to qualitative analysis which combines reflexive thematic analysis with structured early theme development (48). We will use our model of loneliness to generate initial codes based on typologies of loneliness. We will then analyse interview data coding relevant data into these topic summaries. Throughout we will use inductive approaches to review themes and define and name themes which were not present in the codebook. The process of analysis will be iterative by comparing knowledge from the literature with interviews. We will use NVIVO software to facilitate the organisation of codes during analysis. For example, to categorise participants based on severity of loneliness (moderate, severe, very severe).

Analysis will be undertaken by the lead author (JR). The process of familiarisation will begin at begin at the point of interviews with the checking of transcripts. Members of the research team will be consulted in regular biweekly meetings throughout data collection and analysis to discuss

For peer review only - http://bmjopen.bmj.com/site/about/guidelines.xhtml

developing findings. The use of memo notes will be implemented throughout to record decisions on theme development to increase transparency and share findings with wider team.

Public and patient involvement

The DELONELINESS project seeks consultation from a public and patient involvement (PPI) group which includes over 70 adults with an interest in mental health research and commitment to improve inclusivity by advising on race and ethnicity. In October 2022, four members of the group provided feedback on the recruitment methods outlined in this protocol. The group will be consulted throughout the project, specifically to provide feedback on preliminary findings in July 2023 and strategies for dissemination in October 2023.

Ethics and dissemination

Ethical considerations

This study has been approved by Research Ethics Committees at King's College London (reference number: LRS/DP-21/22-33376) and the University of Sussex (reference number: ER/JH878/1). The researcher conducting interviews is skilled in qualitative methods relating to sensitive topics in health and ageing and will adopt an ethical positioning of engagement and mutual respect (36). Regular clinical supervision with a qualified health psychologist will take place to support the interviewer with emotional aspects of conversations.

Time will be taken at the beginning of each interview to establish a rapport with participants and develop an understand of their own personal definition of loneliness. A risk-protocol is in place for if participants were to elicit an emotional response during interviews. With appropriate consent, the risk protocol includes recording participants GP details. In line with GDPR and data protection requirements the study has data handling and de-identification processes in place to maintain the confidentiality of data will be explained to participants involved in the interviews.

Dissemination

The findings will initially be disseminated within the study team via internal reports and meetings attending by all co-authors. The aim will be to share preliminary findings of understandings of how people with varied experienced of loneliness in later life engage with technology including the identification of barriers and facilitators to technology uptake. For example, findings related to participants preferences for placement of sensors will be feedback to the product design workstream, and findings related to signs and symptoms of loneliness will be feedback to the smart textile sensor workstream. For academic dissemination, findings will also be published in peer-reviewed journals and shared at conferences. For public dissemination, we will share findings at meetings and events including webinars run by project partners Housing Learning Improvement Network, and through blogs published via the Campaign to End Loneliness.

Funding statement

This work is supported by the UK Engineering and Physical Sciences Research Council (ESPRC) and the National Institute of Health and Care Research (NIHR) (grant number: EP/W031434/1; EP/W031442/1).

Author contributions

The study concept and design was conceived by WL, SO, YS, AT and FM. FP and MA assisted in refining the study methodology. JR, AT and FM are responsible for data collection. Analysis will be

For peer review only - http://bmjopen.bmj.com/site/about/guidelines.xhtml

undertaken by JR with support from WL, SO, YS, AT, FM, FP and MA. JR prepared the first draft of the manuscript. All authors critically revised the manuscript and approved the submitted version.

Acknowledgments

This research was reviewed by a diverse multicultural Patient and Public Involvement group who have been specially trained to advise on research proposals and documentation through the Race, Ethnicity And Diversity group (READ): a free, confidential service in England provided by the National Institute for Health Research Maudsley Biomedical Research Centre via King's College London and South London and Maudsley NHS Foundation Trust.

Competing interests: None declared.

References

1. Department for Digital Culture Media and Sport. A connected society: a strategy for tackling loneliness [Internet]. 2018 [cited 2022 Dec 16]. Available from: https://www.gov.uk/government/publications/a-connected-society-a-strategy-for-tackling-loneliness

2. Perlman D, Peplau LA. Toward a Social Psychology of Loneliness. 1981;

3. Maes M, Qualter P, Lodder GMA, Mund M. How (Not) to Measure Loneliness: A Review of the Eight Most Commonly Used Scales. International Journal of Environmental Research and Public Health 2022, Vol 19, Page 10816 [Internet]. 2022 Aug 30 [cited 2022 Sep 13];19(17):10816. Available from: https://www.mdpi.com/1660-4601/19/17/10816/htm

4. de Jong Gierveld J, Tilburg TV. The De Jong Gierveld short scales for emotional and social loneliness: tested on data from 7 countries in the UN generations and gender surveys. Eur J Ageing. 2010;121–30.

5. Mansfield L, Victor C, Meads C, Daykin N, Tomlinson A, Lane J, et al. A conceptual review of loneliness in adults: Qualitative evidence synthesis. Vol. 18, International Journal of Environmental Research and Public Health. MDPI; 2021.

6. Cacioppo JT, Cacioppo S. The growing problem of loneliness. The Lancet [Internet]. 2018 Feb 3 [cited 2022 Oct 20];391(10119):426. Available from: http://www.thelancet.com/article/S0140673618301429/fulltext

7. Blakely KK, Baker NR. Loneliness in Community-Dwelling, Older Adults: An Integrative Review. The Journal for Nurse Practitioners. 2022 Aug 4;

8. Courtin E, Knapp M. Social isolation, loneliness and health in old age: a scoping review. Health Soc Care Community [Internet]. 2017 May 1 [cited 2022 Sep 28];25(3):799–812. Available from: https://onlinelibrary.wiley.com/doi/full/10.1111/hsc.12311

9. Donovan NJ, Okereke OI, Vannini P, Amariglio RE, Rentz DM, Marshall GA, et al. Association of Higher Cortical Amyloid Burden With Loneliness in Cognitively Normal Older Adults. JAMA Psychiatry [Internet]. 2016 Dec 1 [cited 2022 Dec 20];73(12):1230–7. Available from: https://jamanetwork.com/journals/jamapsychiatry/fullarticle/2575729

10. Cacioppo S, Capitanio JP, Cacioppo JT. Toward a neurology of loneliness. Vol. 140, Psychological bulletin. 2014. p. 1464–504.

For peer review only - http://bmjopen.bmj.com/site/about/guidelines.xhtml

11. Holt-Lunstad J, Smith TB, Baker M, Harris T, Stephenson D. Loneliness and Social Isolation as Risk Factors for Mortality: A Meta-Analytic Review. Perspectives on Psychological Science. 2015 Mar 16;10(2):227–37.

12. Campaign to end loneliness. The Psychology of Loneliness: Why it matters and what we can do [Internet]. 2020 [cited 2023 Jan 19]. Available from: https://www.campaigntoendloneliness.org/wp-content/uploads/Psychology_of_Loneliness_FINAL_REPORT.pdf

13. Chawla K, Kunonga TP, Stow D, Barker R, Craig D, Hanratty B. Prevalence of loneliness amongst older people in high-income countries: A systematic review and meta-analysis. PLoS One [Internet]. 2021 Jul 1 [cited 2022 Dec 16];16(7):e0255088. Available from: https://journals.plos.org/plosone/article?id=10.1371/journal.pone.0255088

14. Age UK. All the Lonely People: Loneliness in Later Life. 2018.

15. Office for national statistics. Loneliness: What characteristics and circumstances are associated with feeling lonely? 2018;

16. Su Y, Rao W, Li M, Caron G, Arcy CD', Meng X. Prevalence of loneliness and social isolation among older adults during the COVID-19 pandemic: A systematic review and meta-analysis. Int Psychogeriatr [Internet]. 2022 [cited 2022 Dec 13];1–13. Available from: https://doi.org/10.1017/S1041610222000199

17. Vlachantoni A, Evandrou M, Falkingham J, Qin M. The Impact of Changing Social Support on Older Persons' Onset of Loneliness During the COVID-19 Pandemic in the United Kingdom. Gerontologist . 2020 Sep 7;62(8):1147–59.

18. Kitzmüller G, Clancy A, Vaismoradi M, Wegener C, Bondas T. "Trapped in an Empty Waiting Room"—The Existential Human Core of Loneliness in Old Age: A Meta-Synthesis. Qual Health Res. 2018 Jan 1;28(2):213–30.

19. Akhter-Khan S, Prina M, Wong G, Mayston R, Li L. Understanding and Addressing Older Adults' Loneliness: The Social Relationship Expectations Framework. Perspectives on Psychological Science. 2022;

20. Malli MA, Ryan S, Maddison J, Kharicha K. Experiences and meaning of loneliness beyond age and group identity. Sociol Health Illn [Internet]. 2022 [cited 2022 Dec 13]; Available from: https://onlinelibrary.wiley.com/doi/full/10.1111/1467-9566.13539

21. Kharicha K, Manthorpe J, Iliffe S, Davies N, Walters K. Strategies employed by older people to manage loneliness: Systematic review of qualitative studies and model development. Vol. 30, International Psychogeriatrics. Cambridge University Press; 2018. p. 1767–81.

22. Kharicha K, Manthorpe J, Iliffe S, Chew-Graham CA, Cattan M, Goodman C, et al. Managing loneliness: a qualitative study of older people's views. Aging Ment Health. 2021;25(7):1206–13.

23. Tan AJ, Mancini V, Gross JJ, Goldenberg A, Badcock JC, Lim MH, et al. Loneliness Versus Distress: A Comparison of Emotion Regulation Profiles. 2022 [cited 2022 Dec 13]; Available from: https://doi.org/10.1017/bec.2022.18

24. Barjaková M, Garnero A. Risk factors for loneliness A literature review [Internet]. 2022. Available from: https://ec.europa.eu/jrc

For peer review only - http://bmjopen.bmj.com/site/about/guidelines.xhtml

25. Cohen-Mansfield J, Hazan H, Lerman Y, Shalom V. Correlates and predictors of loneliness in older-adults: a review of quantitative results informed by qualitative insights. Int Psychogeriatr [Internet]. 2016 Apr 1 [cited 2022 Sep 7];28(4):557–76. Available from: https://www.cambridge.org/core/journals/international-psychogeriatrics/article/abs/correlates-and-predictors-of-loneliness-in-olderadults-a-review-of-quantitative-results-informed-by-qualitative-insights/EF57448627F1EF5CFD89F83C3F188175

26. Dahlberg L, McKee KJ, Frank A, Naseer M. A systematic review of longitudinal risk factors for loneliness in older adults. Aging Ment Health [Internet]. 2022 [cited 2022 Oct 13];26(2):225–49. Available from: https://www.tandfonline.com/doi/abs/10.1080/13607863.2021.1876638

27. Hutten E, Jongen EMM, Hajema KJ, Ruiter RAC, Hamers F, Bos AER. Risk factors of loneliness across the life span. J Soc Pers Relat. 2022 May 1;39(5):1482–507.

28. Matcham F, Ali S, Hotopf M, Chalder T. Psychological correlates of fatigue in rheumatoid arthritis: A systematic review. Vol. 39, Clinical Psychology Review. Elsevier Inc.; 2015. p. 16–29.

29. Ejlskov L, Wulff J, Bøggild H, Kuh D, Stafford M. Assessing the relative importance of correlates of loneliness in later life. Gaining insight using recursive partitioning. Aging Ment Health [Internet]. 2018 Nov 2 [cited 2022 Sep 28];22(11):1486–93. Available from: https://www.tandfonline.com/doi/abs/10.1080/13607863.2017.1370690

30. Teater B, Chonody JM, Davis N. Risk and Protective Factors of Loneliness among Older Adults: The Significance of Social Isolation and Quality and Type of Contact. Soc Work Public Health. 2021;36(2):128–41.

31. Akhter-Khan S, Au R. Why Loneliness Interventions Are Unsuccessful: A Call for Precision Health. Adv Geriatr Med Res. 2020;

32. Qirtas MM, Zafeiridi E, Pesch D, White EB. Loneliness and Social Isolation Detection Using Passive Sensing Techniques: Scoping Review. JMIR Mhealth Uhealth [Internet]. 2022 Apr 1 [cited 2022 Sep 29];10(4). Available from: /pmc/articles/PMC9044142/

33. Latikka R, Rubio-Hernández R, Lohan ES, Rantala J, Nieto Fernández F, Laitinen A, et al. Older Adults' Loneliness, Social Isolation, and Physical Information and Communication Technology in the Era of Ambient Assisted Living: A Systematic Literature Review. J Med Internet Res 2021;23(12):e28022 https://www.jmir.org/2021/12/e28022 [Internet]. 2021 Dec 30 [cited 2022 Sep 29];23(12):e28022. Available from: https://www.jmir.org/2021/12/e28022

34. Gedam S, Paul S. A Review on Mental Stress Detection Using Wearable Sensors and Machine Learning Techniques. IEEE Access. 2021;9:84045–66.

35. Matcham F, Leightley D, Siddi S, Lamers F, White KM, Annas P, et al. Remote Assessment of Disease and Relapse in Major Depressive Disorder (RADAR-MDD): recruitment, retention, and data availability in a longitudinal remote measurement study. BMC Psychiatry [Internet]. 2022 Dec 1 [cited 2023 Apr 21];22(1):1–19. Available from: https://link.springer.com/articles/10.1186/s12888-022-03753-1

36. Naughton-Doe R, Barke J, Manchester H, Willis P, Wigfield A. Ethical issues when interviewing older people about loneliness: reflections and recommendations for an effective methodological approach. Ageing Soc [Internet]. 2022 Sep 1 [cited 2022 Sep 7];1–19.

For peer review only - http://bmjopen.bmj.com/site/about/guidelines.xhtml

Available from: https://www.cambridge.org/core/journals/ageing-and-society/article/ethical-issues-when-interviewing-older-people-about-loneliness-reflections-and-recommendations-for-an-effective-methodological-approach/A98A61944D6152B43DC68FAA444B3363

37. Campaign to end loneliness. Measuring your impact on loneliness in later life [Internet]. 2015 [cited 2022 Dec 16]. Available from: https://www.campaigntoendloneliness.org/wp-content/uploads/Loneliness-Measurement-Guidance1.pdf

38. Office for National Statistics. Measuring loneliness: guidance for use of the national indicators on surveys [Internet]. 2018 [cited 2022 Dec 16]. Available from: https://www.ons.gov.uk/peoplepopulationandcommunity/wellbeing/methodologies/measuringlonelinessguidanceforuseofthenationalindicatorsonsurveys

39. Hughes ME, Waite LJ, Hawkley LC, Cacioppo JT. A Short Scale for Measuring Loneliness in Large Surveys: Results From Two Population-Based Studies NIH Public Access. Finally, shifting Res Aging. 1999;26(6):655–72.

40. Steptoe A, Shankar A, Demakakos P, Wardle J. Social isolation, loneliness, and all-cause mortality in older men and women. Proc Natl Acad Sci U S A [Internet]. 2013 Apr 9 [cited 2022 Dec 16];110(15):5797–801. Available from: https://www.pnas.org/doi/abs/10.1073/pnas.1219686110

41. Kroenke K, Wu J, Yu Z, Bair MJ, Kean J, Stump T, et al. Patient health questionnaire anxiety and depression scale: Initial validation in three clinical trials. Psychosom Med [Internet]. 2016 Jul 1 [cited 2022 Dec 16];78(6):716–27. Available from: https://journals.lww.com/psychosomaticmedicine/Fulltext/2016/07000/Patient_Health_Questionnaire_Anxiety_and.10.aspx

42. Kroenke K, Spitzer RL, Williams JBW, Löwe B. An Ultra-Brief Screening Scale for Anxiety and Depression: The PHQ–4. Psychosomatics. 2009 Nov 1;50(6):613–21.

43. Beecham J, Knapp M. Costing psychiatric interventions. In: Measuring Mental Health Needs. 2nd ed. Gaskell; 2001. p. 200–24.

44. Chung CCY, Fung JLF, Lui ACY, Chan MCY, Ng YNC, Wong WHS, et al. Client Service Receipt Inventory as a standardised tool for measurement of socio-economic costs in the rare genetic disease population (CSRI-Ra). Nature Scientific Reports [Internet]. 2021 [cited 2022 Dec 16];11(23837). Available from: www.nature.com/scientificreports

45. Pawson R, Tilley N. Realist evaluation . Sage, editor. 1997.

46. Day A, Phelps K, Maltby J, Palmer E, Snell K, Raine D, et al. A realist evaluation of loneliness interventions for older people. Age Ageing [Internet]. 2021 Nov 10 [cited 2022 Dec 21];50(6):2246–53. Available from: https://academic.oup.com/ageing/article/50/6/2246/6376583

47. Pawson R. Causality for beginngers [Internet]. 2008 [cited 2022 Dec 21]. Available from: http://eprints.ncrm.ac.uk/ 245/

48. Braun V, Clarke V. Reflecting on reflexive thematic analysis. Qual Res Sport Exerc Health [Internet]. 2019 Aug 8 [cited 2022 Dec 21];11(4):589–97. Available from: https://www.tandfonline.com/doi/abs/10.1080/2159676X.2019.1628806

For peer review only - http://bmjopen.bmj.com/site/about/guidelines.xhtml

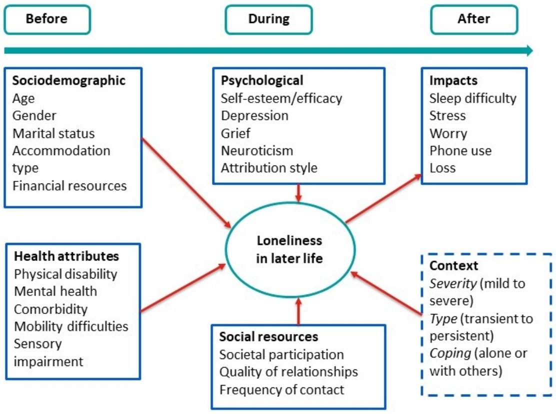

Figure 1

338x190mm (96 x 96 DPI)

Version Number 0.1 31/05/22

SEMI-STRUCTURED INTERVIEW

Ethical Clearance Reference Number: LRS/DP-21/22-33376

Exploring the Psychological Experience of Loneliness

The primary purpose of this interview is to gain insight into the loneliness in older people, and to inform sensor development work being conducted within the DELONELINESS project. The purpose of this interview is threefold:

1) identify the psychological and social parameters of loneliness, prioritised for consideration within a smart system;
2) describe the context and circumstances in which a smart system might be most useful; and
3) identify the most meaningful way of providing information back to individuals, their carers, or heatlthcare/social service providers.

Anonymised quotes may be used in internal reports, external publicity (such as soundbites on the DELONELINESS website), and for research purposes. The interview will be a maximum of 2 hours, and will be recorded for future reference. This guide is designed to provide a structure for interviewers to follow, but does not rule out opportunities to adapt or change the questions, or their order, depending on what the interviewee says.

Interview Phase/Purpose	Questions/Prompts
"Thank you for participation in this interview. I'd like to start by asking you some questions about your experiences of loneliness, either now, or in the past. For all of these questions, please try and specifically think of a time you've felt lonely since your 65th birthday"	
**Definition of loneliness**	1. Can you tell me how you describe loneliness? What does the word mean to you?<br>2. Do you identify with another word? (social connection, social isolation, aloneness, solitude).<br><br>*Prompt*:<br>• Social loneliness (discrepancy between actual and desired quantity and quality of social interactions, includes cultural differences).<br>• Emotional loneliness (absence of meaningful relationships, negative feels can occur ever in close contact with people).<br>• Existential loneliness (sense of separateness from others and wider world, particulary being illness and bereavement).<br>• Relationship-specific loneliness (romantic partner, siblings, children, friends, community).
**Confirming experience of loneliness**	You mentioned in the questionnaire that [insert answer from loneliness measures]. Thinking about these aspects can you tell me:

Version Number 0.1 31/05/22

• Identify when the individual felt lonely	3. Please describe the last time you felt lonely?<br>4. How long did you feel lonely for?<br>*Prompts: Did you feel lonely for weeks or months or years? Or for hours and days? Different before/after COVID?*<br>5. How long ago were you feeling this way, or are you still feeling lonely?<br>6. Did you feel lonely all the time or was it broken up by periods of not feeling lonely?
**Personal experiences of loneliness**	7. When you think about the most significant period of loneliness you've experienced since your 65th birthday, can you describe how it felt?<br><br>*Prompts: Describe anything that comes to mind – your mental health, social life, relationships, physical health…*
**Correlates of loneliness**<br>• Establish the holistic experience of loneliness.	"Thank you for sharing that with me. You mentioned that [insert answer to Q7] was associated with the loneliness you experienced. Can you tell me more about that?<br><br>*Prompt: Did they occur at the same time? Can you describe the experience in more detail? Did X finish when the period of loneliness had ended?*<br><br>Repeat for all the things listed for Q7…
**Precursors to loneliness**<br>• Determine what things might be able to predict loneliness.	Thinking about the most significant period of loneliness you've experienced since turning 65…<br>8. Can you remember anything happened in the weeks, months or years leading up to it which might have contributed to it?<br>*Prompts: Had you had any changes to your usual routine, life events, or diagnoses? COVID-19?*<br><br>"You've mentioned that [insert answer to Q8] happened shortly before your most significant period of loneliness."<br>9. How long, roughly, before you started feeling lonely, did you start to experience this?<br>10. Is there anything that could have stopped [insert answer to Q8] from influencing your loneliness?<br>11. Is there anything else you haven't mentioned so far that could have been associated with the onset of your most significant experience of loneliness?

For peer review only

For peer review only - http://bmjopen.bmj.com/site/about/guidelines.xhtml

Version Number 0.1 31/05/22

	Prompts: Feel free to mention anything, no matter how big or small it might have seemed at the time.
**Implications of loneliness and support received**<br>• Understand what the end of a loneliness event might look like and the care pathways used.	12. Thinking about your most significant period of loneliness, can you tell me more about the aspects of your life it affected?<br><br>*Prompts: Describe anything that comes to mind – your mental health, social life, relationships, physical health…*<br><br>13. How did the loneliness end? Did it finish naturally, or was there an event or intervention which helped it go away?<br>14. If something specific helped it go away, can you explain what happened?<br><br>*Prompts: Did you actively seek help? Who did you speak to? What did they do? If you didn't actively seek help, what triggered the change in your loneliness.*<br><br>15. If your loneliness is ongoing, what do you think would help it go away?
**Final thoughts**<br>• Opportunity to discuss anything else	"Is there anything else about your experience of loneliness which you would like to share before we move onto the next part of the interview?"
**OPPORTUNITY FOR A BREAK IF REQUIRED**	
**The role of technology in measuring loneliness**<br><br>"Thank you for sharing such personal information about your experiences of loneliness. The next part of the interview is going to move on to talk about who technology might be used to help us measure loneliness more effectively."	
**Environment and daily routine**<br>Establishing patterns of activity when lonely and when not lonely	"I would like to move on by asking some questions about your daily routine and your living environment."<br>1. Describe your living environment to us please?<br>2. Which room/furniture in your living environment do you use the most? How long?<br><br>*Prompts: Are you living in a flat or a house? How many rooms are there? Do you have regular house guests?*<br><br>3. Can you describe a typical daily routine for you?<br><br>*Prompts: What time do you tend to wake up? Do you have the same breakfast every day? Do you leave the house every day?*

For peer review only - http://bmjopen.bmj.com/site/about/guidelines.xhtml

	4. Does this routine change much when you're experiencing loneliness?<br>5. [If yes] What tends to change in your daily routine when you're lonely?
**Existing use of technologies**	6. Do you use technology to measure any aspect of your health right now?<br><br>*Prompts: This could be an app on your phone, a website you access regulalry, or some kind of technology that you wear or carry with you to measure something.*<br><br>7. [If yes], please tell me more about it.<br><br>*Prompts: How often do you use it? What works well/favourtie? What works less well/least favourite?*
**Data collection requirements**<br>• Identify priorities for the smart system	"Part of the DELONELINESS study is developing new ways of measuring loneliness, through sensors which might be worn on the body, or integrated into a fabric. These sensors will measure things, such as heart rate, or small movements you make, which might be a useful indicator of loneliness."<br><br>8. Thinking now about a device which you might wear on your body, where on your body would you be willing to wear a device?<br><br>*Prompts: Would you be willing to wear something around your wrist like a watch? Would you prefer something on your ankle, or attached to your chest?*<br><br>9. Wrist: what would make you less willing to wear this device every day? What would make you more likely to wear this device every day?<br>10. Would you be willing to wear the device all day, or would you want to be able to take it off sometimes? Why would you want to take it off?<br><br>Another option might be to have a sensor which is in fabric in your clothes, or in furniture.<br><br>11. If we were to integrate the sensor into fabric in a piece of furniture, what would be the most convenient piece of furniture to use?<br>12. What would make you less willing to use this piece of furniture every day? What would make you more likely to use this piece of furniture every day?

For peer review only - http://bmjopen.bmj.com/site/about/guidelines.xhtml

Version Number 0.1 31/05/22

	13. If we were to integrate the sensor into fabric in an item of clothing, what type of clothing would be most useful?<br>14. What would make you less willing to wear this item of clothing every day? What would make you more likely to wear this item of clothing every day?<br>15. Would you rather have clothing which had a sensor in, or would you rather have a piece of furniture with the sensor in?<br>16. What types of materials or fabric bring you the most comfort at home?
**Loneliness impact on engagement**<br>• Establish whether answer to any of these questions might be different when lonely.	Thinking about the conversation weve just had about your preferences for wearable technologies and textile sensors.<br>17. Do you think any of your opinons or requirements would change if you were feeling lonely?<br>18. If you're currently feeling lonely, do you think any of your opinions or requirements would change if you weren't feeling lonely right now?
**Final thoughts**<br>• Opportunity to discuss anything else	19. Is there anything else about your preferences or requirements for a device which you haven't had an opportunity to mention so far?
**OPPORTUNITY FOR A BREAK IF REQUIRED**	
**1. Data feedback and integration into services**<br><br>"Thank you for giving us an insight into your living environment and your preferences for how we could be measuring loneliness using sensors. We're now going to move onto the final part of the interview, which focuses on what we should do with the data we collect. We might be able to collect data about a wide range of things, such as your sleep, movements and stress levels."	
**Data recipients**<br>• Who should receive it?	20. How useful would this data be for you, your family or caregivers and your GP to receive?<br>21. Is there any other person or service this data could be sent to to help improve your quality of life?
**Implications of data**<br>• What the data would mean	22. If this data were sent to you…what benefits are there to having this kind of information available?<br><br>*Prompts: Might this data change your daily routines, or prompt you to do something different?*<br><br>23. What limitations might there be to having this kind of information available?<br>[repeat questions for family or caregiver and GP if relevant]

For peer review only - http://bmjopen.bmj.com/site/about/guidelines.xhtml

**Data requirements and actions**<br>• How data might be received	24. What would you do if we were to alert you that you were at risk of becoming lonely?<br>25. If this data were sent to you…how would you want to receive it? How often?<br>26. What would you hope a carer or family member/GP would do if we were able to alert them that you were at risk of becoming lonely?
**Final thoughts**<br>• Opportunity to discuss anything else	27. Is there anything else about your what we could do with the information we collect you haven't had an opportunity to mention so far?

For peer review only

For peer review only - http://bmjopen.bmj.com/site/about/guidelines.xhtml
